# Silent spontaneous posterior uterine rupture of a prior caesarean delivery at 36 weeks of gestation

**DOI:** 10.1186/s12884-019-2180-3

**Published:** 2019-01-11

**Authors:** Shao Hui Chen, Xiu Ping Du

**Affiliations:** Department of Obstetrics and Gynaecology, Women and Children’s Hospital of Shanxi Province, Taiyuan, 030001 People’s Republic of China

**Keywords:** Uterine rupture, Pregnancy, Caesarean section

## Abstract

**Background:**

In caesarean section patients, the spontaneous rupture of the posterior wall of the uterus is extremely rare, with nonspecific signs and symptoms being present. Perinatal and maternal morbidity and mortality are high.

**Case presentation:**

A 28-year-old woman at 36 + 6 weeks of gestation presented with mild uterine contractions and developed a sudden abdominal distension. An emergency laparotomy was performed, and the posterior wall of the uterus had ruptured. A baby boy was born.

**Conclusion:**

Silent uterine rupture is very rare and easy to ignore due to nonspecific clinical symptoms, unexplained haemoglobin reduction and haemoperitoneum, but these features caution us to more closely consider uterine rupture in patients.

## Background

Uterine rupture is an obstetric complication that causes significant maternal and foetal morbidity and mortality [1]. Silent uterine rupture is very difficult to diagnose, as the clinical features of uterine rupture, including abdominal pain, vaginal bleeding, maternal hypovolemic shock, or haemorrhage may be absent [2]. We present the spontaneous rupture of the posterior wall of the uterus at 36 weeks of gestation; the uterine rupture was found during the operation.

## Case presentation

**Fig. 1:**
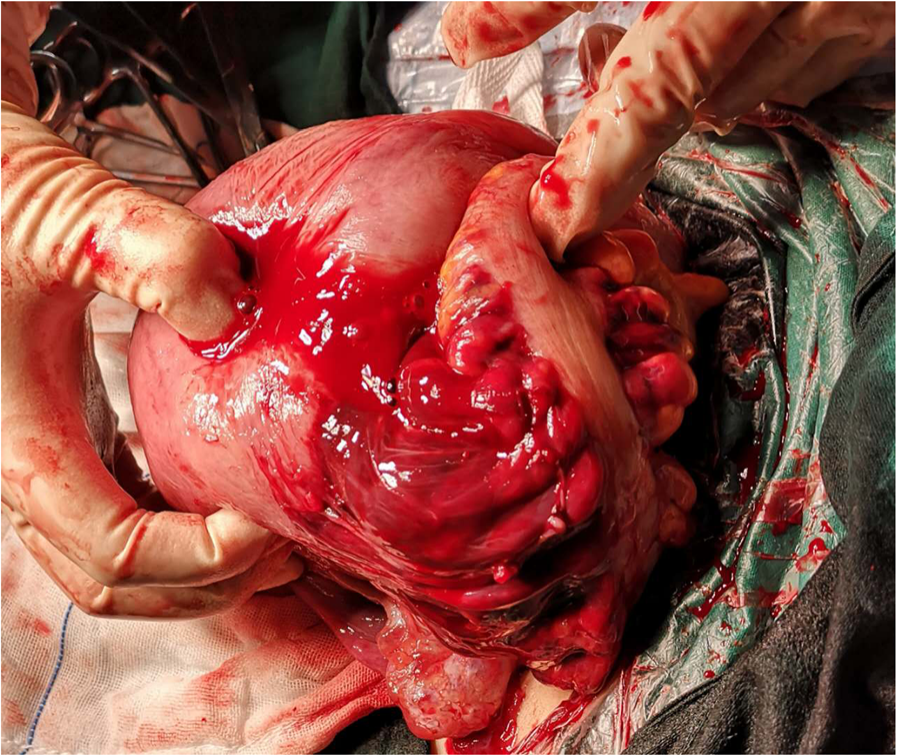
Defect of the posterior wall of the uterus adhered to the bowel, haematoma on mesentery, active bleeding on the defect

**Fig. 2:**
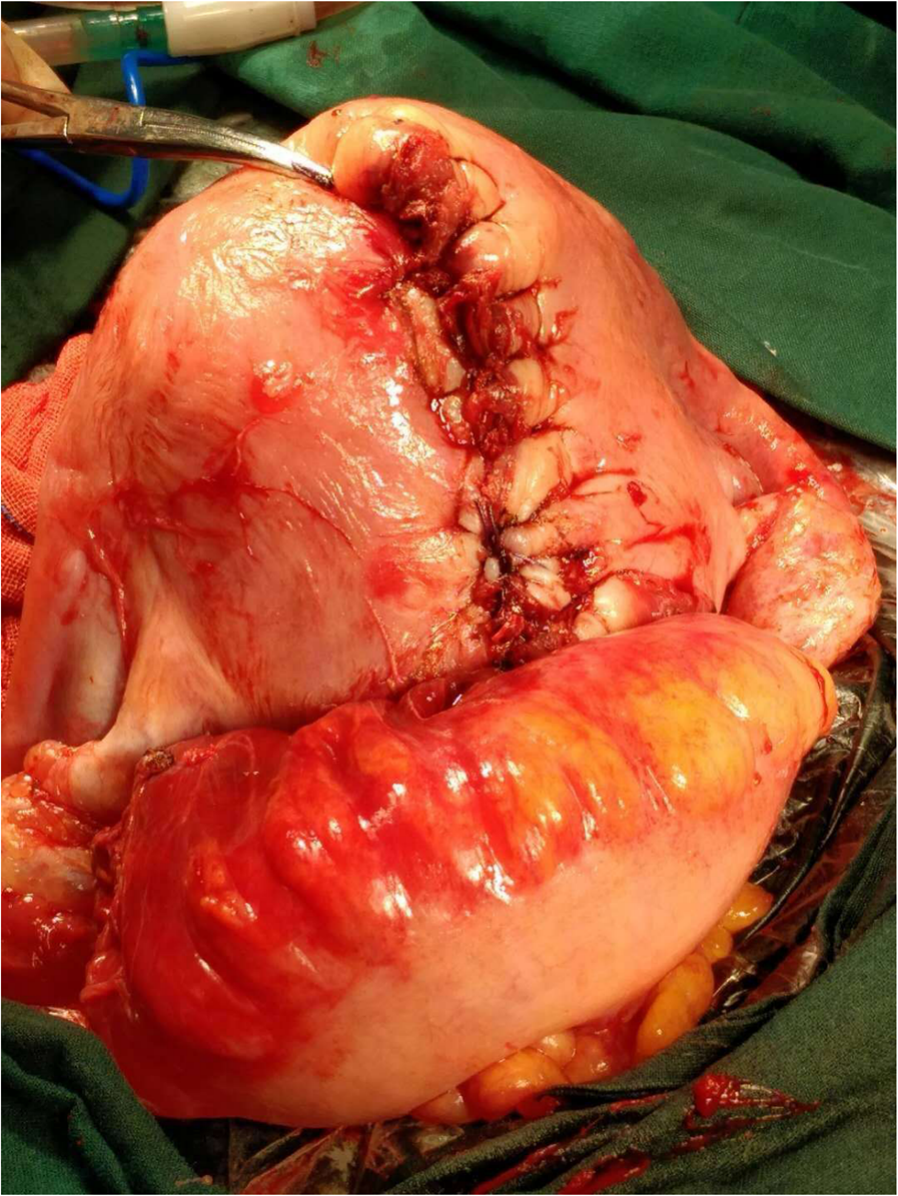
A 12-cm-long tear was shown after separating the adhesion between the intestinal tube and uterus. Continuous suture and embedding were performed

A 28-year-old woman, gravida 3 para 1, had a medical termination of a miscarriage at seven weeks, with no dilation and curettage, in 2008. In 2015, a baby was delivered by caesarean section in the breech position, weighing 3900 g. She had no significant past medical history, and her antenatal care had been uneventful. On August 9, 2018, at 19:15, she was admitted to our hospital due to a pregnancy of 9+ months and irregular contractions for 4+ hours. Periodic uterine contractions occurred every 6–8 min. The patient was not accompanied by abdominal pain or vaginal bleeding and had intermittent term after contractions. Clinical examination showed that her body temperature was 36.7 °C, blood pressure was 102/65 mmHg, pulse rate was 100 bpm, and oxygen saturation was 100%. Blood tests showed mild leucocytosis (16.61 × 10^9^/L), normal platelet count, normal coagulation test, and haemoglobin of 102 g/L. Vaginal examination showed the cervix was tightly closed; no vaginal bleeding or fluid was found. The ultrasonography indicated that the foetal head was located above the uterine cavity, the foetal size was consistent with the gestational age, the placental position was normal, and the scar thickness of the previous caesarean section was approximately 0.2 cm. Uterine contractions declined after admission. During admission, the patient was clinically and biochemically stable, and daily cardiotocograms showed a reassuring foetal heart rate pattern. Because of the patient’s progressive anaemia (blood tests revealed a slow decline in haemoglobin to 93 g/L, 87 g/L) and sudden increasing abdominal pain, ultrasound was used but did not show ruptured abdominal fluid. An urgent laparotomy was performed and revealed a massive haemoperitoneum caused by the rupture of the uterine posterior wall. A haemoperitoneum with approximately 1 liter of blood was recovered. The lower uterine segment was intact and not ruptured. A boy with a body weight of 2900 g was delivered. Apgar scores were 9 at 1 min and 10 at 5 min. The amniotic fluid was clear, the placental was completely delivered, and no placental abruption occurred. The patient’s uterus was closed in two layers. After removing the blood and clots, a 12 cm-long tear in the posterior wall and active bleeding from the uterine rupture were found. Uterine tissue adhered to the bowel (see Fig. 1). After separation of the adhesions between the bowel and the uterine wall, two layer of uninterrupted stitches restored the uterine integrity, and interrupted stitches closed the mesentery defect (see Fig. 2). It was suspected that future conceptions would be dangerous, so bilateral tubal ligation was performed at the same time, under the permission of the patient and the patient’s family member. Our patient’s uterine and pelvis showed no abnormalities and, particularly, no evidence of endometriosis. Inspection of her liver showed no rupture. The placenta was sent for pathological examination. Syntocinon (oxytocin) (Ma an Mountain Company, China, SFDA approval number: H34020474) was administered intravenously. The operation was uncomplicated, and the estimated total blood loss was 2500 ml. Ten units of blood and 400 ml of blood plasma were transfused. The patient’s postoperative course was regular, and she was discharged 6 days later.

## Discussion

Spontaneous rupture of the posterior wall of the uterus in pregnancy is rare and potentially a catastrophic event for both the mother and the foetus [3, 4]. Nonspecific signs and symptoms lead to misdiagnosis and delayed treatment. In this case, no predisposing factors, classic signs and symptoms, including decreased foetal heart rate, uterine contraction, abdominal pain, changes in the station of the presenting part, bleeding or shock were found. The patient felt only uterine contraction aggravations and abdominal swelling. We performed an urgent laparotomy based on the previous caesarean delivery history in breech presentation. Both the patient and the newborn were fortunate to have a good outcome.

In 2011, Stefano Uccella [5] wrote a review of spontaneous pre-labour uterine rupture in a primigravida. Some risks in those cases included a history of uterine surgeries, such as caesarean section or myomectomy, uterine damage due to trocar insertion, uterine perforation and other risk factors, such as uterine anomaly, uterine curettage, uterine diverticula, and Ehlers-Danlos syndrome. The patient had only a history of caesarean section, with no other uterine operations, but the rupture site was not found in the uterine scar. She had no other risk factors.

Le-Ming Wang [6] reported a spontaneous uterine rupture on the posterior wall due to placenta percreta. In this case, the placenta was located on the right lateral and anterior wall of the uterus so that its occurrence should not be related to placenta factors. Unscarred uterus multiparity is one of the most important factors in uterine rupture. The stretching, tearing or bruising of repeated childbirth makes the uterine wall very weak, so the chances of rupture increase with every subsequent pregnancy. The patient had a medical termination of a missed miscarriage at seven weeks and a caesarean section. It was not clear if this rare event of spontaneous rupture may be attributed to the weakening of the uterine wall.

Traditionally, spontaneous rupture of the posterior wall of the uterus is rare, and the rupture is often easily covered by the intestinal loop and omentum so that some minor symptoms are ignored. Ultrasonography plays a critical role in diagnosing uterine rupture based on the demonstration of a myometrial defect associated with intraperitoneal and extraperitoneal haemorrhage [7]. In this case, we failed to find extraperitoneal haemorrhage. However, it is important to maintain a high index of suspicion for uterine rupture in women presenting with some or all of these features, regardless of any known risk factor [8]. Prompt recognition of uterine rupture, early diagnosis and expeditious recourse to laparotomy are critical to influencing perinatal and maternal morbidity.

## Conclusion

Silent uterine rupture, especially that occurring in the posterior wall, is very rare and easy to ignore due to nonspecific clinical symptoms. Haemoglobin reduction and haemoperitoneum in patients caution us to closely consider uterine rupture.
